# Understanding Drug Permeability in *Pseudomonas aeruginosa*

**DOI:** 10.3390/life15111705

**Published:** 2025-11-04

**Authors:** Ishan Ghai

**Affiliations:** Department of Life Sciences and Chemistry, Constructor University, 28759 Bremen, Germany; ishanghai@live.com

**Keywords:** antibiotics, *Pseudomonas aeruginosa*, gram-negative bacteria, outer membrane, cell envelope, porins, permeability, antimicrobial resistance, membrane transport

## Abstract

*Pseudomonas aeruginosa* is a Gram-negative bacterium that poses a serious threat to patients with weakened immunity, cystic fibrosis, severe burns, or those in hospitals. Its ability to resist antibiotics comes largely from its outer membrane, which blocks drug entry. This means higher doses are often needed, raising the risk of side effects. To design new treatments, researchers need drugs that not only bind strongly to bacterial targets but also cross this tough membrane. Unfortunately, there are few reliable methods to directly measure how easily drugs pass through the *Pseudomonas aeruginosa* cell envelope. Recent advances, such as electrophysiology-based flux studies, have started to reveal how different antibiotics particularly β-lactams move through porin channels. These studies show large differences in permeability, but the findings remain scattered. What is missing is a unified dataset that captures permeability under varied conditions. Such a resource would clarify how porin structures influence drug entry and help chemists design better compounds. This review brings together current knowledge on drug permeability in *Pseudomonas aeruginosa*, with a focus on electrophysiological and related methods. This review highlights the need for standardized approaches that generate consistent and comparable data. A comprehensive “permeability atlas” could guide the development of new antibiotics by fine-tuning molecular properties like size, charge, and lipophilicity, ultimately improving porin passage and restoring treatment effectiveness against this challenging pathogen.

## 1. Introduction

Advanced experimental techniques, such as single-channel electrophysiology and molecular simulations, have begun to shed light on transport dynamics at the microscopic level [1,2,3,4,5,6,7,8,9]. However, despite these advances, a comprehensive dataset that accurately characterizes the permeability of the outer membrane under various conditions remains elusive and insufficient [4,6,8,9,10]. This gap in our understanding not only hinders the development of new antibiotics but also limits our ability to design drugs that can bypass or exploit the unique properties of the *Pseudomonas aeruginosa* cell envelope [4,9,11]. The pathogen is known to cause a wide range of infections—from pneumonia and urinary tract infections to bloodstream infections and sepsis—each of which poses significant treatment challenges due to its multidrug-resistant phenotype [12,13,14,15,16,17]. Particularly in patients with compromised immune systems, the rapid progression of these infections can lead to serious outcomes, making timely and effective antibiotic therapy critical [13,14,16,18,19]. In addition, the adaptability of *Pseudomonas aeruginosa* has led to the emergence of strains resistant to last-resort antibiotics such as carbapenems, further complicating treatment strategies and exacerbating the global antimicrobial resistance crisis [16,20,21,22,23,24,25,26].

Understanding the molecular architecture of the *Pseudomonas aeruginosa* outer membrane is central to overcoming these challenges [20,21,27,28,29,30]. Unlike the inner membrane, which is relatively more permeable to small molecules, the outer membrane acts as a gatekeeper, selectively filtering out potentially harmful compounds while allowing essential nutrients to pass through [31,32,33,34,35]. The selective permeability of the outer membrane in Gram-negative bacteria is primarily governed by the presence of outer membrane proteins also known as porins or channels [6,36]. These proteins assemble into water-filled channels that facilitate the passive diffusion of small hydrophilic molecules across the membrane [7,35,37,38,39,40].

**Figure 1:**
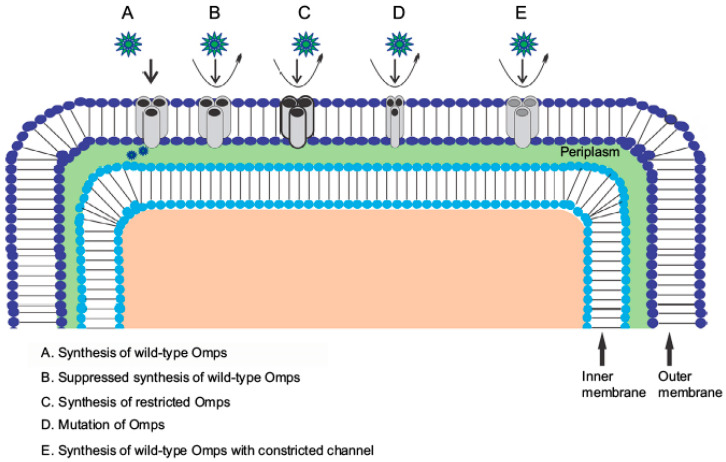
Antibiotic-resistance mechanism associated with Omp modification. Antibiotic β-lactam molecules are represented by green stars, and Omps as trimers by gray cylinders. The width of the straight arrows represents the level of β-lactam penetration via Omps. The curved arrows exemplify the uptake failure/reduce uptake occurring with the following: B: decrease in the level of wild-type Omps expression; C: expression of restricted-channel Omps; D: mutation or modification of the functional properties of a porin channel; and E: synthesis of modified Omps with significant constriction. Abbreviation: Omps, outer membrane proteins. Note: Copyright ©2017. Dove Medical Press. * Note This figure is a modified and updated version of the author’s previously published work Infection and Drug Resistance 2017:10 261–273 Adapted Originally published by and used with permission from Dove Medical Press Ltd. [36].

The specificity and regulation of these channels are critical, as even minor structural changes can drastically alter the permeability properties of the membrane [4,7,9,38,41,42]. For example, variations in porin expression or changes in their structure can significantly reduce the influx of antibiotics, thereby contributing to the bacterium’s resistance profile [43,44,45,46,47,48]. Further understanding of the outer membrane of *Pseudomonas aeruginosa* as a dynamic, asymmetric bilayer whose adaptive modifications regulate permeability to balance nutrient influx with protection from external threats is important [6,20,30,49,50,51,52,53,54]. In response to antibiotic and environmental stress, the bacterium remodels its lipid A moiety—via two-component systems such as ParR–ParS and PhoP–PhoQ, activating The PagP enzyme and the arn operon modify the bacterial outer membrane by adding palmitoyl chains and 4-amino-4-deoxy-L-arabinose, which reduces surface negative charge and helps repel cationic drugs [30,54,55,56,57]. Concurrently, surface-exposed loops of major porins (e.g., OprD, OprG, and members of the Occ family) undergo partial or complete deletion, constricting the pore entrance and disrupting key substrate-docking interactions to diminish carbapenem and other small-molecule uptake [20,43,50,58,59,60,61] (Figure 1). Antibiotic-resistance mechanisms are associated with outer membrane protein modifications [11,35,36,49,52,62,63]. Finally, single-point mutations within the narrow “eyelet” region of porin channels introduce steric hindrance or local charge shifts; well-characterized examples in OprD slow diffusion of imipenem and meropenem without compromising essential nutrient passage [7,43,59,63,64,65,66,67,68]. Through these concerted lipid and protein alterations, *Pseudomonas aeruginosa* enacts a robust, tunable barrier—fine-tuning its outer membrane architecture to thwart antibiotic entry while preserving viability [10,69,70,71,72,73,74].

The development of novel antibiotics that can effectively target *Pseudomonas aeruginosa* must therefore consider the intricacies of this outer membrane barrier [12,37,65,75,76]. Traditional drug-discovery approaches, which often focus on the biochemical interactions between antibiotics and their intracellular targets, may overlook the critical step of membrane permeation [8,77,78]. A shift in focus is needed—one that emphasizes the role of membrane transport in determining the overall efficacy of antimicrobial agents [4,6,8,9,35,36,52]. By integrating insights from molecular biology, biophysics, and computational modeling, researchers can develop more sophisticated strategies to enhance drug delivery across the outer membrane [4,8,9]. For example, quantitative permeability measurements—whether obtained from planar-bilayer conductance experiments, electrophysiology flux measurements, molecular dynamics (MD) simulations, or other methodologies—allow one to define clear physicochemical ‘windows’ in which antibiotic scaffolds must fall to traverse specific porin channels efficiently [1,4,7,8,9,10,49,77,79]. By plotting apparent permeability coefficients (Papp) against molecular weight and polar surface area, one can empirically observe that compounds under ~600 Da with a polar surface area of 60–100 Å^2^ achieve the highest flux through OprD-type porins [20,50,58,65]. Likewise, mapping Papp as a function of cLogD reveals that mildly cationic molecules (global charge +1 to +2 at physiological pH) optimally exploit the negatively charged constriction zone; this insight directly informs the choice and placement of basic amine functionalities on candidate scaffolds [20,29,43,50,58,59,64,65]. Furthermore, distance-dependent charge pairing can be tuned: measuring how flux varies with the inter-cationic distance (e.g., two positively charged centers spaced ~12–15 Å apart) guides linker design to match the eyelet geometry of the pore [50,58,59,80]. Taken together, these quantitative permeability–structure relationships enable medicinal chemists to rationally adjust scaffold dimensions, dipole moment, and charge distribution—rather than relying on trial and error—to maximize translocation rates while preserving target engagement and overall antibacterial potency [7,10,15,81,82].

This review explores the role of membrane protein transporters in facilitating antibiotic movement across the outer membrane of *Pseudomonas aeruginosa*. Furthermore, this study highlights key scientific advancements in understanding how various solutes, including antibiotics, are taken up by the bacterium (Table 1 and Table 2). A deeper insight into outer membrane permeability (Table 3) can support future antibiotic development by leveraging computational data to decode bacterial transport mechanisms and design drugs with enhanced permeability [4,7,8,9,10,11,36,83].

Furthermore, building on the detailed examination of porin structure and function, the next critical step involves quantifying their role in antibiotic transport across the outer membrane [1,3,11,35,79,81,83,100]. While transport and MD studies provide valuable insights into porin architecture, understanding the dynamics of molecular influx requires advanced experimental and computational approaches [3,52,81,83,100]. The following section focuses on quantitative methodologies for measuring permeability through porin channels—specifically, planar-bilayer conductance experiments, electrophysiology flux measurements, and MD simulations—and highlights how such data can inform rational antibiotic design to help overcome the selective barrier of *Pseudomonas aeruginosa* [81,91,93]. Other experimental approaches, while valuable, are not covered in detail here.

### 1.1. Study Selection Criteria

This review was developed through a comprehensive literature survey of peer-reviewed research articles that investigate *Pseudomonas aeruginosa* outer membrane permeability and porin-mediated antibiotic transport. Databases including PubMed, Scopus, and Web of Science were systematically searched using combinations of keywords such as “*Pseudomonas aeruginosa*”, “porins”, “outer membrane”, “permeability”, “antibiotic influx”, and “molecular dynamics simulations”. Studies were included if they provided quantitative or mechanistic insights into drug permeation, porin structure–function relationships, or electrophysiological and flux-based permeability measurements. Priority was given to works presenting original experimental data, supported by computational modeling or high-resolution structural analysis. Review papers, duplicate reports, or studies lacking clear methodological descriptions were excluded. The final selection emphasizes representative and high-impact publications that collectively describe the current understanding of drug permeability across the *Pseudomonas aeruginosa* outer membrane. Furthermore, this work builds on and extends the author’s previous contributions in the field [11,36,52,83].

### 1.2. Understanding and Quantifying Outer Membrane Permeability in Pseudomonas aeruginosa

The successful development of novel antibiotics against Gram-negative bacteria continues to be profoundly impeded by the formidable barrier posed by their outer membrane [1,4,6,8,9,10]. In *Pseudomonas aeruginosa*, this barrier is primarily constituted by a complex array of specialized porins that tightly regulate the uptake of hydrophilic molecules, including many antibiotic classes [3,16,34]. Due to the inherently low permeability of these porins, higher extracellular concentrations of antibiotics are often required to achieve therapeutic intracellular levels, inadvertently raising the risk of systemic toxicity and off-target effects [82]. As a result, a deep understanding—coupled with quantitative assessment—of the permeability barrier imposed by these porins is indispensable for rational antibiotic design [6,9].

At the heart of this permeability challenge lies the sophisticated architecture of the *Pseudomonas aeruginosa* outer membrane, which is interspersed with a diverse set of porins predominantly from the OccD and OccK families [34,87,88,90]. These β-barrel channels, embedded within an asymmetric lipid bilayer, serve as fine tuned gateways that allow the selective passage of small, hydrophilic molecules. As summarized in Table 1 and Table 2, critical porins include OccD1 (OprD), OccD3 (OpdP), and members of the OccK family (OccK1–OccK8), each exhibiting distinct substrate preferences [20,32,34,43,50,58,59,64,65,67,71,80,84,87,88,90,101]. For instance, OprD is widely recognized as the principal conduit for carbapenems such as imipenem and meropenem [43,79,84,85,94,98,99], while OprO and OprP selectively mediate Fosfomycin uptake [79,85,94,98,99]. Studies consistently underscore that these porins not only facilitate antibiotic influx but also significantly shape the bacterium’s resistance landscape by modulating access to intracellular targets [50,72,79,85,99].

Importantly, these porins do not function merely as passive molecular sieves. Their permeability properties are governed by a constellation of structural and physicochemical features—including pore diameter, electrostatic charge distributions, ion selectivity filters, dynamic gating mechanisms, and specific amino acid interactions at constriction zones [28,44,49,50,58,65,93,98,102,103,104]. Even minute alterations in these parameters—whether due to mutations, regulatory changes, or environmental stress responses—can lead to substantial shifts in antibiotic uptake rates, with direct consequences for drug efficacy and resistance profiles [50,58,61]. This necessitates not only structural characterization but also rigorous quantification of antibiotic translocation through these channels [6,9].

Indeed, recent advances have enabled detailed quantification of influx rates, bridging the gap between structural insight and functional impact [9]. Techniques such as single-channel electrophysiology [103,105,106] and electrophysiology reversal potential measurements have systematically measured permeability coefficients and turnover rates for various antibiotics across specific porins [1,35,37,79,83,100]. For example, flux measurements reveal that OprO can transport Fosfomycin at rates nearing 280 molecules per second under a 10 μM concentration gradient, whereas OprP exhibits markedly lower transport efficiency under comparable conditions [79]. MD simulations further complement these experiments by mapping free-energy landscapes of antibiotic passage, elucidating why certain structural motifs enhance or impede translocation [85,94,98,99].

Taken together, these insights illustrate how the interplay of porin architecture, substrate specificity, and dynamic regulation not only underpins the adaptability of *Pseudomonas aeruginosa* to hostile environments—including antibiotic pressure—but also poses a significant challenge to therapeutic intervention [58,64,79,80,84,85,94,98,99,107]. By systematically integrating quantitative influx data with high-resolution structural and functional analyses, researchers can better decipher how porins contribute to complex resistance phenotypes [49,51,77,80]. Such a comprehensive understanding is vital for exploiting these channels in drug design, guiding the development of antibiotics that are precisely tailored to navigate this intricate permeability barrier [4,7,77]. Future investigations must continue to prioritize coupling structural elucidation with direct measurements of transport efficiency to pave the way for overcoming this critical obstacle in treating *Pseudomonas aeruginosa* infections [49,50,51,80,91,98].

## 2. Experimental Approaches to Understanding the Role of Outer Membrane Porins in *Pseudomonas aeruginosa* Towards Antibiotic Influx

Measuring the transport of antibiotics across the outer membrane has involved multiple experimental strategies. Each method provides unique insights while also complementing the limitations of others.

### 2.1. Electrophysiological Measurements

Electrophysiology is an exceptionally informative and versatile method for probing the activity of individual ion channels in biological membranes. In these experiments, porins—integral membrane proteins that form channels—are reconstituted into an artificial planar lipid bilayer [105,106,108,109]. A controlled transmembrane voltage is then applied across this synthetic membrane [9,10,104,105,106,110]. The application of this voltage induces an ion current that flows through the porin channels, and the resulting electrical signals are recorded with high precision [4,77,93,98,109].

This technique enables researchers to obtain detailed insights into several key aspects of channel function. For instance, by monitoring the ion current at the single-channel level, scientists can measure the channel’s conductance, which indicates how easily ions traverse the pore [1,37,61,79,81,83,100]. Additionally, electrophysiological recordings reveal the channel’s ion selectivity by demonstrating which specific ions are preferentially conducted. This is crucial for understanding the biochemical and biophysical properties of the channel [1,9,10,37,61,79,81,83,100,104,110].

Another significant advantage of this method is its ability to capture the dynamics of channel gating [16,61,89,111]. Gating refers to the mechanisms by which channels open and close in response to stimuli. By analyzing the fluctuations in the ion current, researchers can elucidate the kinetics of these gating events, providing a window into the regulatory mechanisms that control channel activity [16,61,89,111]. This information is invaluable, as it helps in pinpointing the rate-limiting steps involved in the transport process [9,10,16,61,89,104,110,111].

Furthermore, the precise measurements obtained through electrophysiology allow scientists to determine the maximum size of molecules that can be accommodated by a particular channel [9,10,31,49,51,81,112]. This aspect is especially important when evaluating how small molecules, such as drugs and antibiotics, pass through the bacterial outer membrane. By understanding these limitations, researchers can better assess the efficiency of molecular transport and identify potential bottlenecks [1,9,10,31,34,41,49,51,81,82,112,113].

Overall, electrophysiological techniques offer a detailed and quantitative approach to studying ion channels [1,9,10,31,34,41,49,51,81,82,112,113]. The data gathered from these experiments not only advance our understanding of membrane transport at the molecular level but also inform the design of therapeutic agents by highlighting the structural and functional parameters that govern channel activity. This comprehensive insight into channel behavior is essential for developing strategies to overcome barriers in drug delivery and for designing novel interventions against multidrug-resistant pathogens [34,77,81,82,112].

Further, MD simulations have become an indispensable tool in the study of antibiotic translocation through outer membrane porins, offering atomistic resolution that complements traditional electrophysiological assays [3,4,7,38,49,77,81,114,115,116]. While electrophysiology provides real-time measurements of ion conductance through individual channels, it lacks the spatial detail needed to resolve transient molecular interactions. MD simulations bridges this gap by explicitly modeling all atoms, including antibiotics, water molecules, and ions, thereby enabling the direct observation of hydrogen bonding, electrostatic contacts, and van der Waals interactions with pore-lining residues [4,38,61,90,99,100,117,118]. These simulations have revealed multiple translocation pathways: (i) simple diffusion with transient binding, in which antibiotics pause at charged or polar “hotspot” residues before continuing along the pore axis; (ii) pore dehydration-induced entry, where displacement of the hydration shell around the constriction zone reduces the free-energy barrier for entry; and (iii) multi-step hopping, where bulkier antibiotics traverse the pore by sequentially occupying energetically favorable sub-sites identified through potential of mean force (PMF) profiles [38,39,68,115,117,119]. Despite being conducted under idealized conditions—such as constant temperature, pressure, and simplified bilayer compositions—MD simulations provide robust quantitative insight into antibiotic–porin interactions that are difficult to capture experimentally [38,49,61,77,91,93,94,117,119,120,121]. By mapping free-energy landscapes along translocation coordinates, simulations elucidate the molecular basis of antibiotic permeability and resistance, particularly in cases involving structural mutations such as the G103K substitution in Neisseria PorB, which narrows the pore and impedes drug passage [73]. These insights not only explain reduced permeation rates observed in resistant strains but also guide rational antibiotic design by identifying physicochemical properties—such as charge distribution, conformational flexibility, and hydration penalties—that optimize outer membrane uptake. Thus, MD simulations serve as a powerful platform for dissecting the microscopic determinants of antibiotic transport and resistance in Gram-negative pathogens [28,49,60,88,98,116,119,122].

### 2.2. Flux Measurements

Quantitative analysis of flux rates, as presented in Table 3, reveals distinct transport efficiencies among porins in *Pseudomonas aeruginosa*. For instance, OprO shows high permeability for fosfomycin, with a flux of about 28 molecules per second at a 1 μM concentration, increasing nearly tenfold to roughly 280 molecules per second under a 10 μM gradient. In comparison, OprP transports fosfomycin at much lower rates—≤1 molecule per second at 1 μM and around 2.2 molecules per second at 10 μM. Similarly, OprE exhibits limited permeability across several molecules. Ceftazidime passage through OprE is recorded at ≤1 molecule per second at 1 μM. Furthermore, the transport of cefotaxime and carbenicillin via OprE is minimal, with fluxes of ≤1 molecules per second at 1 μM. Additionally, small charged molecules such as sodium glutamate and arginine chloride displayed similar permeability through OprE, with flux rates of ≤1 molecule per second at 1 μM, increasing modestly at the higher gradient [81]. Overall, these measurements establish a robust quantitative framework for comparing the efficiency of various porins and assessing how alterations in antibiotic structure might enhance permeation [9,10,104,111]. The observed variations in selective permeability underscore the complexity of antibiotic uptake in *Pseudomonas aeruginosa* and highlight potential avenues for optimizing drug-delivery strategies targeting these bacterial channels.

### 2.3. Integrating Data: A Comprehensive Picture of Antibiotic Influx

Integrating diverse experimental approaches is essential to fully understand the role of outer membrane porins in *Pseudomonas aeruginosa* and their influence on antibiotic permeability [79,80,85]. No single technique can provide a complete picture of the molecular processes involved; instead, it is the convergence of data from multiple disciplines—biophysics, computational modeling, microbiology, and structural biology—that yields meaningful insights into porin-mediated transport [4,6,7,8,9,10,51,110,122].

Techniques such as single-channel electrophysiology, liposome-swelling assays, and molecular dynamics (MD) simulations, flux measurements offer complementary perspectives on porin function. Electrophysiology, for example, measures real-time ion flux through individual porins and can quantify conductance, selectivity, and gating behaviors [1,4,6,7,9,51,79,112,113,115,123]. This method has been crucial in identifying subtle functional shifts in porins such as OccK1 or OprD that correlate with resistance phenotypes [34,87,88,90]. In parallel, MD simulations provide atomic-level views of how antibiotics traverse porin channels. They allow researchers to track transient binding events, hydrogen bonding patterns, and energy barriers associated with molecular movement through constriction zones [34,49,87,90,116,119].

Quantitative flux data, such as those presented in Table 3, further reinforce the functional differences among porins. For instance, fosfomycin shows high permeability through OprO, with flux reaching ~280 molecules/second at 10 µM, whereas OprP transports the same molecule at less than 10% of that rate. Such discrepancies highlight how even structurally similar porins can vary dramatically in transport efficiency due to minor differences in pore geometry and charge distribution [79].

A particularly illustrative case is the carbapenem class of antibiotics. Studies have demonstrated that OprD serves as the main entry route for imipenem and meropenem [75,84]. In resistant clinical isolates, downregulation or mutation of OprD correlates directly with elevated minimum inhibitory concentrations (MICs) [75,84,96,101]. For example, substitutions in OprD’s constriction loop or loss-of-function mutations have been observed in isolates from ICU patients, correlating with treatment failure [75,84,96,101]. When OprD is absent or nonfunctional, alternative porins such as OprF or OprE may provide limited uptake, but they cannot fully compensate, leading to therapeutic challenges [3,20,32,43,59,64,65,70,72,84,101].

These insights have not remained academic. Medicinal chemists have applied this permeability knowledge to redesign existing scaffolds. Modified carbapenems have been engineered with reduced molecular weight, optimized polar surface area, and strategically positioned positive charges to enhance their translocation through porins like OprD [20,32,43,59,64,65,70,72,101]. Structure–activity relationship (SAR) studies have used computational permeability indices, derived from porin-specific assays, to prioritize compounds for development. Some of these efforts have even led to clinical candidates now being evaluated for multidrug-resistant Gram-negative infections [20,29,32,43,58,59,64,65,70,72,80,101].

Beyond drug design, integrated data enable predictive modeling. By correlating permeability coefficients with structural features and clinical resistance profiles, researchers can now simulate the impact of porin loss or mutation on antibiotic efficacy [43,59,64]. These models help identify which drug–porin combinations are likely to be successful and under what physiological conditions. Moreover, they offer a valuable platform for testing adjuvant strategies, such as efflux pump inhibitors or porin-inducing agents, that could restore antibiotic susceptibility [10,63,67,116].

Thus, integrating biophysical, computational, and microbiological data provides a powerful framework for understanding and overcoming the permeability barrier in *Pseudomonas aeruginosa* [4,6,8,9,10]. This systems-level approach not only advances basic science but also has direct translational relevance, informing the design of more effective antibiotics capable of penetrating the outer membrane and maintaining activity in resistant strains.

## 3. Discussion: Porin Modifications and Their Role in Antibiotic Resistance

Antibiotic resistance arising from the loss or modification of porins is a well-documented phenomenon [6,7,8,9,10,104,111]. Bacterial cells often undergo regulatory changes or mutations in porin genes that lead to reduced expression or structural alterations of these channels [9,42,61,104,113,114]. Such modifications can drastically decrease antibiotic influx, thereby conferring resistance. Studies have shown that both regulatory events and mutations in the porin genes—and even in the broader regulatory cascades controlling envelope permeability—can lead to decreased porin production or altered channel architecture [9,38,42,44,63,81].

This phenomenon is not unique to *Pseudomonas aeruginosa*. Similar adaptive responses have been observed in species like *Escherichia coli*, *Enterobacter aerogenes*, *Enterobacter cloacae*, and *Klebsiella pneumoniae* during antibiotic treatment [6,9,38,42,44,49,54,63,66,80,81,124]. Continuous exposure to antibiotics applies a selective pressure that favors successive modifications in porin expression, effectively reducing drug influx and thereby increasing resistance. In extreme cases, complete loss of porin expression renders bacteria virtually impermeable to β-lactams and other antimicrobials, representing a robust adaptive mechanism to counteract antibiotic attack [6,20,41,70].

Mutations that occur near or within the constriction region of porin channels are particularly detrimental [43,63,68,120,125]. For example, studies of porin mutants isolated from patients undergoing chemotherapy have demonstrated that specific conformational changes in the pore lumen can significantly impede β-lactam translocation, leading to enhanced bacterial resistance. Site-directed mutagenesis experiments further underscore the importance of particular amino acid residues in the constriction region; substitutions at key positions often result in altered ion selectivity and reduced transport efficiency [7,43,44,66,93,98]. These findings highlight the crucial role of the constriction zone in maintaining the delicate balance between nutrient uptake and protection against harmful substances [58,60,61].

Electrophysiological techniques, such as planar lipid bilayer assays, have been instrumental in characterizing these changes at a molecular level By examining the structural and functional properties of porin channels, researchers can directly observe the impact of specific mutations or deletions [43,63,68,120,125]. For instance, deletion of internal loop regions in porins like OccK1 has been shown to modify gating dynamics, thereby altering conductance and the overall flux rate [58,60,61]. This type of analysis is critical for identifying new targets for antimicrobial strategies, as it points to specific structural features that could be modulated to enhance antibiotic uptake [36,66,113,114].

The direct role of outer membrane porins in antibiotic transport has also been highlighted in studies that explore the interaction of antibiotics with the membrane proteins [10,49,61,62,81,110,112,121]. Techniques such as conventional electrophysiology have revealed that the presence of antibiotics can induce distinct current changes, reflective of binding events or temporary pore blockage. These molecular-level interactions are key to understanding the transport mechanism [10,49,61,62,81,110,112,121]. They also provide a basis for designing antibiotic molecules that can either avoid or exploit these interactions to improve translocation across the membrane [80,82,91,122].

Targeting porins to overcome resistance is a promising strategy. By designing molecules that can better interact with or even modulate the function of these channels, it may be possible to increase antibiotic influx despite adaptive resistance mechanisms [9,10,60,114].

However, fully elucidating the molecular underpinnings of antibiotic passage through outer-membrane porins demands the seamless integration of diverse methodologies [9,10]. High-resolution electrophysiology pinpoints how transient drug–porin contacts modulate single-channel conductance and dwell time distributions, while stopped-flow liposome-swelling assays yield quantitative permeability coefficients (P_app) for reconstituted porin–lipid systems [6,8,9,10,29]. Mass spectrometry-based uptake measurements then correlate these permeability metrics with actual intracellular antibiotic accumulation across strains differing in porin expression [8,9,29]. Complementing experiments, accelerated MD simulations reveal the microsecond-scale motions of critical gating residues—most notably the acidic–basic hydrogen-bond pair in loop L3—and delineate the dynamic salt-bridge and hydrogen-bond networks at the constriction “eyelet” that govern open-to-closed transitions [8,9,29,58,60,61]. By mapping the free-energy landscape of these gating events, researchers can identify interaction hotspots whose targeted mutation or ligand engagement markedly enhances antibiotic flux—insights that feed directly into rational scaffold design, optimizing molecular dimensions, dipole alignment, and charge distribution to overcome the permeability barrier [4,10,38,58,60,61,80,81,117].

## 4. Conclusive Remarks: The Path Forward

The intricate interplay between *Pseudomonas aeruginosa*’s outer membrane architecture and its antibiotic-resistance mechanisms presents a formidable challenge in modern antimicrobial therapy. This review underscores that the bacterium’s low-permeability outer membrane, governed by specialized porins such as OprD and members of the OccD/OccK families, serves as a critical frontline defense against antibiotics [20,29,34,43,50,59,64,65,67,72,80,87,88,90]. These porins exhibit remarkable structural diversity and substrate specificity, enabling selective transport of nutrients while restricting the influx of antimicrobial agents. For instance, carbapenems like imipenem and meropenem may rely heavily on OprD for entry [20,43,70,72,80,84], while fosfomycin might exploits phosphate-specific channels such as OprO and OprP [79,85,98,99]. However, mutations, reduced expression, or structural modifications in these porins—documented extensively in clinical isolates—directly correlate with elevated resistance, highlighting the evolutionary adaptability of *Pseudomonas aeruginosa* [63,66,68,98].

The compilation of experimental and MD simulations data (Table 1 and Table 2) reveals a critical gap: despite advances in single-channel electrophysiology and structural biology, a comprehensive quantitative framework for antibiotic influx through these channels remains elusive [11,36,52,83]. Current methodologies, while informative, often fail to account for dynamic factors such as porin regulation under stress, lipid bilayer interactions, or the synergistic effects of efflux pumps [30,55,56]. This gap impedes the rational design of antibiotics capable of bypassing or exploiting porin-mediated transport [4,8,9,10].

### 4.1. Key Implications for Antibiotic Development

#### 4.1.1. Porin-Targeted Drug Design

Structural insights into porin–substrate interactions (e.g., OprD’s β-barrel architecture or OccK1’s anion selectivity) could guide the development of antibiotics with optimized molecular dimensions and charge profiles to enhance uptake [32,59,64,65,67,70].

#### 4.1.2. Dynamic Permeability Modeling

Integrating computational simulations with empirical data on porin gating behaviors and conductance states (e.g., OccK5’s multi-state dynamics) may enable predictive models of antibiotic influx under varying physiological conditions [34,87,88,90].

#### 4.1.3. Combination Therapies

Pairing novel antibiotics with adjuvants that transiently disrupt membrane integrity or upregulate porin expression could counteract resistance mechanisms [34,49,68,77].

### 4.2. Limitations and Future Perspectives

Despite the substantial progress achieved in elucidating the permeability characteristics of *Pseudomonas aeruginosa*, several important limitations remain that restrict the comprehensive understanding and translation of current findings into clinical applications.

1. Variability in Experimental Approaches

A major limitation lies in the heterogeneity of experimental methodologies used to study porin-mediated transport. Techniques such as electrophysiology, liposome swelling, fluorescence-based assays, and mass spectrometry often employ different membrane compositions, ionic strengths, and temperature conditions. These variations lead to inconsistencies in permeability measurements and make it difficult to compare results across different laboratories. Furthermore, the lack of universally accepted reference compounds and calibration standards adds another layer of complexity in correlating permeability coefficients between studies [4,8,9,10,29].

2. Limited Physiological Relevance of In Vitro Models

Most existing studies are conducted under simplified in vitro conditions that do not fully replicate the native bacterial envelope or its environmental context. In the physiological setting, *P. aeruginosa* exists in complex biofilm communities and experiences dynamic changes in osmotic pressure, pH, and nutrient availability—all of which influence porin expression and functionality. Current artificial membrane systems or reconstituted porin assays, while informative, fail to capture these adaptive regulatory mechanisms. Consequently, permeability data derived from such systems may not accurately represent in vivo drug transport behavior [4,8,9,10,30,51,55,122].

3. Underrepresentation of Efflux–Permeability Interplay

While this review primarily focuses on the permeability barrier, antibiotic accumulation in *P. aeruginosa* is also profoundly affected by efflux pump systems such as MexAB–OprM, MexXY, and MexCD–OprJ [10,14,63,67,101,116]. The interplay between limited influx through porins and active efflux removal determines the effective intracellular concentration of antibiotics. However, very few studies integrate both aspects simultaneously. A more holistic approach—combining permeability assays with efflux quantification—would provide a realistic understanding of how drugs accumulate within the bacterial cell [10,14,59,63,64,65,67,79,87,90,94,98,99,101].

4. Structural and Dynamic Uncertainty in Porin Models

Although crystallographic and cryo-EM structures of major porins (e.g., OprD, OprF, OprP, and members of the Occ family) have greatly improved structural insight, they often represent static snapshots of proteins. In contrast, porins in living cells undergo continuous conformational changes influenced by transmembrane potential, ion concentration, and ligand binding [3,32,43,59,64,65,80,81,91,93,94,99]. Molecular dynamics (MD) simulations help bridge this gap but depend heavily on the choice of force fields, simulation timescales, and membrane parameters, which may introduce biases. Experimental validation of these computational predictions remains limited [3,10,14,59,63,64,65,67,79,80,81,85,87,88,90,94,98,99,101].

5. Lack of Standardized Permeability Databases

Currently, there is no comprehensive or publicly available database compiling quantitative permeability data for *P. aeruginosa* porins under standardized conditions [4,9,10]. The absence of such a resource hinders cross-comparison, meta-analysis, and the development of predictive models for drug design. Establishing a curated “permeability atlas” with unified data reporting standards—including pore conductance, molecular descriptors, and flux rates—would represent a valuable step forward for the scientific community [6,8,9,10].

6. Translational Gap between Basic Research and Drug Discovery

While biophysical and computational studies have yielded valuable mechanistic insights, the integration of permeability parameters into actual drug design pipelines remains limited [8,9,11,36,78,83]. Medicinal chemists often prioritize target affinity and pharmacokinetic properties over membrane penetration efficiency. To overcome this translational barrier, permeability indices derived from porin-specific studies should be systematically incorporated into lead optimization stages to guide scaffold modification and molecular tuning [9,78].

### 4.3. Future Directions

Addressing these limitations requires a multidisciplinary and standardized research framework.

Future work should aim to:

- Develop integrated experimental platforms combining electrophysiology, flux assays, and live-cell uptake measurements under physiologically relevant conditions.
- Create high-throughput screening systems that evaluate permeability across multiple porins simultaneously using microfluidic or biosensor-based technologies.
- Employ machine learning and AI models trained on existing permeability data to predict antibiotic uptake and identify physicochemical features that favor porin passage.
- Explore dynamic regulatory mechanisms of porin expression under clinical stressors such as antibiotic exposure, oxidative stress, and host immune factors.
- Encourage collaborative databases and open-access repositories that standardize permeability reporting across research groups.
- Foster translational collaborations between structural biologists, microbiologists, and medicinal chemists to design next-generation antibiotics optimized for both target affinity and membrane penetration.

In conclusion, although significant advances have been made in understanding the permeability barrier of *Pseudomonas aeruginosa*, current knowledge remains fragmented due to methodological variability, structural uncertainty, and limited integration with drug discovery. Overcoming these challenges will require standardized, data-driven, and cross-disciplinary strategies that translate molecular insights into clinically effective antimicrobial solutions.

## Figures and Tables

**Table 1:** The table below provides a summary of each study’s focus, the porins analyzed, the methodologies employed, and the key findings, expanding on and updating the author’s previous work [11,36,52]. Please note: The author has made every effort to ensure accurate citations and data. Any errors are unintentional and beyond the author’s control. The table is illustrative and not exhaustive.

Structural Insights and Functional Dynamics	Antibiotic Class Investigated	Porin(s) Examined	Principal Findings
Meropenem permeation through the outer membrane [84]	Meropenem	OprD, OprF, OprE	Meropenem uptake in *Pseudomonas aeruginosa* occurs via alternative porins (OprF/OprE) when OprD is absent, unlike imipenem.
Imipenem resistance associated with amino acid alterations porin in *Pseudomonas aeruginosa* clinical isolates [70]	Imipenem, Meropenem	OprD	This study investigates OprD mutations and expression levels in imipenem-resistant *Pseudomonas aeruginosa* from Ardabil hospitals, highlighting diverse resistance mechanisms.
Fosmidomycin uptake via phosphate-specific channels [85]	Fosmidomycin	OprO, OprP	Elucidated the mechanism of fosmidomycin transport through phosphate-specific porins
Characterization of single-channel properties [34]	NA	OccK8	This study detailed single-channel behavior and conductance of OccK8 and analyzed the effects of Arginine, Glycine, and Glutamic Acid.
Analysis of conductance and gating in the OccD series [86]	NA	OccD1—OccD6	Documented a broad conductance range, multiple gating transitions, and cation selectivity across these channels
Investigation of carboxylate interactions within the channel [27]	NA	OccD1—OccD6, OccK1—OccK7	Identified interactions between carboxylate groups and basic residues (arginine/lysine) that influence uptake
Examination of multi-state gating dynamics [87]	NA	OccK1—OccK7	Revealed distinct one-, two-, and three-open sub-states and established anion selectivity linked to constriction zone residues
Impact of internal loop deletion on channel behavior [88]	NA	OccK1	Demonstrated that removal of the constriction loop significantly alters gating properties
Thermodynamic characterization of channel gating [33]	NA	OccK1	Quantified the activation parameters for loop-deletion-induced transitions in channel activity
Influence of ion concentration on gating transitions [89]	NA	OpdK	Determined that variations in ion levels modulate the gating kinetics of the channel
Structural insight into substrate specificity [50]	NA	OprD	Revealed an 18-stranded β-barrel architecture with a narrow pore, critical for selective substrate transport
Role of surface loops in antibiotic translocation [58]	Imipenem	OprD	Demonstrated that specific extracellular loop regions are crucial for imipenem passage
Dynamics of amino acid substrate movement [80]	Imipenem, Meropenem	OccD1 (OprD)	Provided insight into the structural dynamics and natural substrate translocation through the pore
Uptake kinetics of carbapenem antibiotics [82]	Imipenem, Meropenem	OccD3 (OpdP)	Documented the transport rates of imipenem and meropenem across the channel
Mechanism of tricarboxylate and citrate uptake [44]	NA	OccK5	Clarified the role of OccK5 in the uptake of key metabolites such as isocitrate and citrate
Variability in gating behaviors among porin channels [90]	NA	OccK5	Identified diverse gating properties and conductance states within OccK5
Role in temocillin permeation [15]	Temocillin	OccK1, OccK2	Confirmed the contribution of these porins to temocillin entry into the cell
Energetics of ion selectivity [2,91]	NA	OprP	Established the energetic profile for the selective transport of phosphate, sulfate, chloride, and potassium ions
Critical role of acidic residue in binding [92]	NA	OprP	Highlighted the importance of residue D94 in phosphate binding and selectivity
Contribution of a central basic residue [93]	NA	OprP	Demonstrated that arginine R133 is vital for defining ion transport properties
Determining key constriction determinants [94]	NA	OprP, OprO	Identified essential constriction residues that affect substrate specificity
Reduced porin expression in resistance [21]	Imipenem, Meropenem	OprD	Linked decreased OprD levels to carbapenem heteroresistance
Correlation between porin levels and antibiotic MIC [64]	Imipenem, Meropenem	OprD	Showed that diminished porin expression elevates carbapenem MICs in clinical isolates
Quantification of OprD in resistant strains [20]	Imipenem, Meropenem	OprD	Documented lower OprD transcript levels in carbapenem-resistant isolates
Altered permeability and susceptibility profiles [95]	Imipenem, Meropenem	OprD	Revealed discrepancies in carbapenem susceptibility related to changes in outer membrane permeability
In vitro evaluation of novel β-lactam combinations [96]	Ceftazidime, Avibactam Ceftolozane, Tazobactam	OprD	Demonstrated activity of ceftazidime-avibactam and ceftolozane-tazobactam against meropenem-resistant isolates
Identification of in-frame deletions in clinical isolates [59]	NA	OprD	Detected unique in-frame deletions in OprD among clinical isolates
Variability of membrane protein dominance in resistance [67]	Imipenem, Meropenem	OprD	Observed shifts in membrane protein profiles in imipenem-resistant isolates
Whole-cell assay for permeability relationships [29]	Imipenem, Meropenem	OprD	Characterized structure–permeation correlations for novel carbapenem analogues
Effects of specific amino acid substitutions [72]	NA	OprD	Determined that substitution at codon 170 correlates with increased resistance
Impact of single residue changes on resistance [71]	Imipenem, Meropenem	OprD	Demonstrated that individual amino acid alterations significantly affect carbapenem susceptibility
Consequences of incapacitating mutations [43]	Imipenem, Meropenem	OprD	Showed that severe mutations and decreased expression contribute to high-level resistance
Survey of porin presence in resistant isolates [97]	Imipenem, Meropenem	OprD	Confirmed widespread occurrence of altered OprD in 70 carbapenem-resistant isolates
Double mutations altering ion specificity [98]	NA	OprP, OprO	Demonstrated that double mutations can invert specificity between phosphate and diphosphate transport
Structural features underpinning amino acid transport [3,81]	Ceftazidime Hydrate, Cefotaxime, Carbenicillin	OccK8	Defined substrate-specific transport mechanisms for amino acids via OccK8
Permeation of Fosfomycin through the Phosphate-Specific Channels OprP and OprO of *Pseudomonas aeruginosa* [99]	Fosfomycin, Fosmidomycin	OprP and OprO	Fosfomycin uses specific channels (OprP and OprO) to enter resistant *Pseudomonas aeruginosa* more effectively than fosmidomycin. Exploiting channel selectivity could improve antibiotic uptake in Gram-negative bacteria.
Probing transport of fosfomycin through substrate specific membrane proteins [79]	Fosfomycin	OprO, OprP	Fosfomycin exhibits high permeability through OprO and OprP porins, making it a promising alternative for treating *Pseudomonas aeruginosa* infections.

Note: Copyright ©2017. Dove Medical Press. * Note This table is a modified and updated version of the author’s previously published work Infection and Drug Resistance 2018:11 523–530 and Infection and Drug Resistance 2017:10 261–273 Adapted Originally published by and used with permission from Dove Medical Press Ltd. [36,52].

**Table 2:** Compilation of studies on outer membrane porins in *Pseudomonas aeruginosa*. This table provides an extensive, study-by-study account of research into the structure, function, and permeability characteristics of *Pseudomonas aeruginosa* outer membrane porins. It includes details of the antibiotic classes or specific molecules examined, the porins analyzed, the methodologies employed, and the principal findings reported in the original publications. This comprehensive dataset builds upon and expands the thematic summary presented in Table 1, presenting the same core studies in a more detailed and organized format to enhance scientific clarity. It serves as a detailed reference resource for researchers interested in porin-mediated transport, antibiotic influx, and associated resistance mechanisms. Note: The author has made every effort to ensure accurate citations and data. Any errors are unintentional and beyond the author’s control. The table is illustrative and not exhaustive.

Major Topic	Antibiotic Class/Molecule	Porins Examined	Principal Findings	
Structural Insights	NA	OccK8, OccD1-D6, OprP, OprO, OprD	Characterization of β-barrel architectures, pore diameters, constriction zones, and surface loops critical for substrate specificity. Multi-state gating and charge-selective filters.	[2,27,33,34,50,86,87,88,89,91,92,93,94,98]
Functional Dynamics	NA	OccK1–K7, OccD1–D6, OpdK	Detailed conductance ranges, gating transitions, anion/cation selectivity, and effects of loop deletions or point mutations on permeability.	[27,33,86,87,88,89]
Porin Modifications and Resistance Mechanisms	Imipenem, Meropenem	OprD	Diverse OprD mutations, in-frame deletions, and reduced expression linked to elevated carbapenem MICs and heteroresistance in clinical isolates.	[20,21,29,43,50,58,59,64,67,70,71,72,80,95,96,97]
Transport Mechanisms of Nutrients/Ions	NA	OprP, OprO, OccK5	Mechanistic insights into phosphate, sulfate, chloride, potassium, isocitrate, and citrate uptake; crucial role of constriction residues and charged residues.	[2,44,79,91,92,93,94,98,99]
Antibiotic Uptake Studies	Imipenem, Meropenem, Temocillin	OprD, OccK1, OccK2, OprF, OprE	Carbapenem transport is mainly via OprD; alternative uptake via OprF/OprE when OprD is absent. OccK porins contribute to temocillin entry.	[3,15,20,21,29,43,58,64,67,70,71,80,81,82,84,95,96,97]
Fosfomycin/Fosmidomycin Studies	Fosfomycin, Fosmidomycin	OprP, OprO	High permeability of fosfomycin via OprO (~280 molecules/s) compared to OprP (~2.2 molecules/s), exploiting phosphate-specific channels.	[79,85,99]
Whole-Cell Permeability Correlations	Imipenem, Meropenem	OprD	Reduced OprD levels directly correlate with increased MICs. Structure–permeability relationships established for novel carbapenems.	[29,64,96]
Novel β-lactam Combinations	Ceftazidime-Avibactam, Ceftolozane-Tazobactam	OprD	Showed activity against meropenem-resistant isolates with altered OprD profiles.	[96]

**Table 3:** Reported turnover rates for molecules/s at a given concentration gradient for different outer membrane proteins with different substrates. The values are approximated, and for detailed calculations, please see the research publications mentioned with each value [79,81]. Antibiotics are shown in one column on the right, followed by reported Gram-negative bacterial species, recalculated flux rates in molecules/s at 1 μM for comparison purposes only, and approximate values and reported flux molecules/s at the specific mentioned gradient. Note: The author has made every effort to ensure accurate citations and data and numbers. Any errors are unintentional and beyond the author’s control. The table is illustrative and not exhaustive. Adapted from [83].

Molecules	Omps	Bug	Recalculated Flux at 1 μM Molecules/s	Reported Flux Rate Molecules/s at the Specific Mentioned Gradient
Fosfomycin	OprO	*Pseudomonas aeruginosa*	28	≈280 at gradient 10 μM [79]
	OprP	*Pseudomonas aeruginosa*	≤1	≈2.2 at gradient 10 μM [79]
Ceftazidime	OprE	*Pseudomonas aeruginosa*	≤1	≈0.4 a t gradient 10 μM [81]
Cefotaxime	OprE	*Pseudomonas aeruginosa*	≤1	≈0.1 at gradient 10 μM [81]
Carbenicillin	OprE	*Pseudomonas aeruginosa*	≤1	0.04 at gradient 10 μM [81]
Sodium Glutamate	OprE	*Pseudomonas aeruginosa*	≤1	≈0.6 at gradient 10 μM [81]
Arginine Chloride	OprE	*Pseudomonas aeruginosa*	≤1	≈0.1 at gradient 10 μM [81]

## Data Availability

No new data were created or analyzed in this study. Data sharing is not applicable to this article.
